# Une maladie immunoproliférative de l'intestin grêle révélée par une invagination intestinale aigue: à propos d'un cas

**DOI:** 10.11604/pamj.2014.17.12.3512

**Published:** 2014-01-14

**Authors:** Amal Bennani, Kaoutar Znati, Salima Rezzouk, Hicham Bouhadouti, Khalid Maazaz, Affaf Amarti

**Affiliations:** 1 Service d'Anatomie Pathologique, CHU Hassan II, Fès, Maroc; 2 Service de Chirurgie Viscérale, CHU Hassan II, Fès, Maroc

**Keywords:** Maladie immunoproliférative, intestin grêle, invagination intestinale, immunoproliferative disease, small intestine, intussusception

## Abstract

La maladie immunoproliférative de l'intestin grêle IPSID est un lymphome rare qui se développé à partir du système lymphoïde associé aux muqueuses au niveau de l'intestin grêle. Sa révélation par une invagination intestinale aigue est exceptionnelle et n'a jamais été rapporté dans la littérature auparavant. Nous rapportons le cas d'un patient de 65ans, admis aux urgences dans un tableau d'invagination intestinale aigue. Le scanner abdominal a mis en évidence masse grêlique à paroi concentrique avec incarcération du segment mésentérique au sein de la lésion fortement évocatrice d'une invagination intestinale. Le patient a été opéré et a bénéficié d'une résection iléale emportant le boudin d'invagination. L'examen histologique de la masse a été en faveur d'une maladie des chaines alpha transformée en un lymphome B diffus à grandes cellules. Les auteurs rapportent un cas rare d'une IPSID révélée par une invagination intestinale aigue et à travers cette observation, mettent en relief les principaux aspects cliniques, histologiques, thérapeutiques de cette entité avec une revue de la littérature.

## Introduction

La maladie immunoproliférative de l'intestin grêle (IPSID) également appelé maladie des chaînes lourdes alpha est un lymphome qui touche le système des immunoglobulines A exocrine de la muqueuse et préférentiellement celui de l'intestin grêle et s'accompagne de la sécrétion d'une chaine lourde alpha. Sa révélation par une invagination intestinale aigue est exceptionnelle et n'a jamais été rapporté dans la littérature.

## Patient et observation

**Figure 1:**
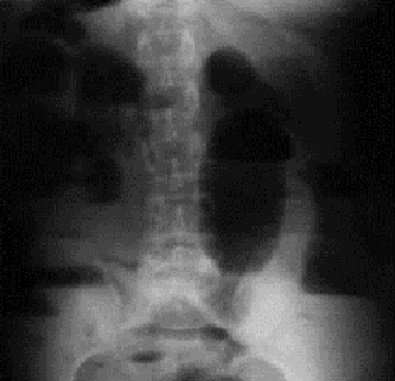
Abdomen sans préparation montrant de multiples niveaux hydro-aériques de type grêliques

**Figure 2:**
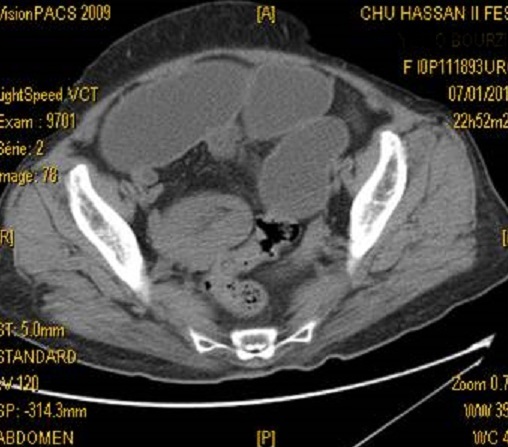
Scanner abdominal en coupes axiales montrant l'image en cocarde correspondant à l'invagination

**Figure 3:**
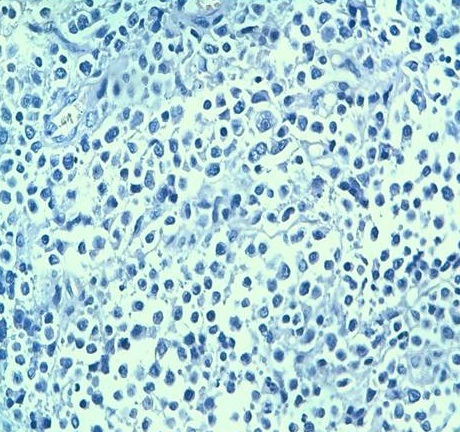
Prolifération de cellules de grande taille formant des clusters au sein de cellules de petite taille à différenciation plasmocytaire(HES X 200)

**Figure 4:**
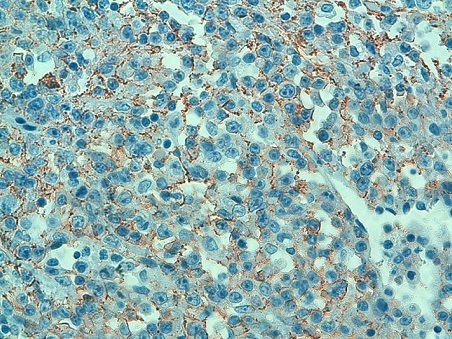
Expression du CD20 par les cellules tumorales

Il s'agit d'un patient âgé de 65 ans, admis au service des urgences dans un tableau d'occlusion intestinale aigue. Le patient rapporte la notion de douleurs abdominales similaires depuis un mois. L'examen clinique a révélé une distension abdominale avec une matité à la percussion, et au toucher rectale une ampoule rectale vide. Une radiographie de l'abdomen sans préparation a mis en évidence de multiples niveaux hydro-aériques grêliques (Figure 1). L’échographie abdominale a mis en évidence une masse digestive pelvienne hétérogène mesurant environ 8 cm de diamètre. Un scanner abdominal complémentaire a montré une masse grêlique à paroi concentrique avec incarcération du segment mésentérique au sein de la lésion fortement évocatrice d'une invagination intestinale (Figure 2). Le patient a été opéré et a bénéficié d'une résection iléale emportant le boudin d'invagination avec une anastomose termino-terminale. L'examen macroscopique de la pièce opératoire a mis en évidence une masse de 7 cm de diamètre exoluminale arrondie, homogène et friable à la coupe qui s'invagine à l'intérieur d'un segment iléal. L'examen histologique a retrouvé prolifération lymphomateuse faite de petites cellules avec différenciation plasmocytaire nette, intéressant toutes les couches de la paroi grêlique et arrivant jusqu'au niveau de la séreuse. Par endroit on notait la présence de cellules lymphoïdes de grande taille immunoblastiques et centroblastiques formant des nappes diffuses et parfois des clusters de 5 à 10 cellules (Figure 3). Une étude immunohistochimique a été réalisée et a montré une forte expression du CD20 par les cellules tumorales (Figure 4). Devant cet aspect morphologique et immunohistochimique le diagnostic retenu est celui d'une maladie des chaines alpha transformée en un lymphome B diffus à grandes cellules. Un bilan d'extension réalisé s'est révélé négatif.

## Discussion

Les lymphomes digestifs constituent la première localisation extra-ganglionnaire des lymphomes et sont, dans l′immense majorité des cas, de type non hodgkinien [1]. Parmi ces lymphomes on ditingue les IPSID (immuno-proliferative small intestinal diseases) qui sont des proliférations diffuses du système lymphoïde B du tube digestif caractérisés par une atteinte diffuse du grêle sans intervalle de muqueuse saine [2, 3].

Ce lymphome affecte les jeunes des deux sexes entre 15 et 30 ans mais il peut atteindre les enfants et les personnes âgées [4]. La majorité des cas recensés provient du bassin méditerranéen et du moyen orient mais de nombreux cas ont été observés chez les malades originaires de l'Europe de l'est, et de l'ex URSS et de l'Amérique centrale et du sud [4]. Le seul dénominateur commun de tous ces malades étant la mauvaise hygiène et le niveau socio-économique bas comme c'est le cas pour notre patient. Lecuit et al ont prouvé grâce à la technique de PCR l'association entre le Campylobacter jéjuni et l'IPSID [5]. La toxine cytoléthale du C. jejuni engendre des cassures double-brin de l'ADN qui favorisent le développement de cellules B mutées, différenciées en cellules plasmocytaires aberrantes produisant des chaînes lourdes tronquées. L'absence de région variable à la surface des cellules B mutées explique qu'elles échappent au système immunitaire, avec pour conséquence une prolifération responsable du développement d'IPSID [6].

L'IPSID se manifeste habituellement par une malabsorption associée à une entéropathie exsudative [7]. Elle peut se manifester également à l'occasion de douleurs abdominales, ou de vomissements. A un stade tardif, la maladie peu prendre une allure tumorale avec une véritable masse abdominale responsable d'une urgence chirurgicale telle que l'invagination intestinale aigue comme c'est le cas de notre patient.

Sur le plan biologique, l'immunofixation des protides sanguins révèle une protéine sérique anormale dans 40 à 100% des cas, qui correspond à des chaînes lourdes alpha sériques. Sa détection est plus importante au stade des IPSID de bas grade, plus rare au niveau des cellules immunoblastiques (stade plus avancé de la maladie).

Un bilan endoscopique doit être réalisé pour mettre en évidence l’étendue des lésions et pour obtenir le diagnostic histologique [8, 9].

La vidéocapsule endoscopique [10] est une technique non invasive et performante, grâce à laquelle l'intestin grêle est le segment du tube digestif le mieux étudié avec une sensibilité meilleure et une rentabilité diagnostique de 50-70%. Des biopsies multiples, étagées doivent être réalisées: fixées dans du formol pour une étude immunohistochimique et de biologie moléculaire, la congélation peut être recommandée (dans le cadre d'essais cliniques).

La stadification utilisée est celle d'Ann Arbor, modifiée par Musshoff pour le tube digestif. Dans la classification de l'OMS 2008, les IPSID correspondent aux LMNH de phénotype B de la zone marginale du mucosa-associated lymphoid tissue (MALT) extraganglionnaires. Dans la classification des lymphomes gastro-intestinaux répertoriés par ISAACSON, ils correspondent aux LMNB du MALT de faible degré de malignité, de type méditerranéen (maladie des chaînes ∝essentiellement).

Galian et al. avaient établi des critères qui classent les IPSID en trois stades selon le grade de malignité [7]. Aux stades A et B, l′intestin grêle est macroscopiquement normal, en dehors d′un épaississement des plis qui sont semés de petits nodules. Au stade C, une ou plusieurs tumeurs ulcérées et/ou un épaississement pariétal étendu sont visibles, des sténoses ou des fistules internes sont possibles.

Sur le plan histologique le stade A se manifeste par une prolifération dense de plasmocytes matures ne franchissant pas la muscularis mucosae. Les villosités sont plus ou moins raccourcies et élargies, le revêtement épithélial est peu altéré, les cryptes sont rares, écartées et atrophiques. Le Stade C est caractérisé par la présence d′un lymphome immunoblastique avec différenciation plasmocytaire. Et c'est le cas de notre malade. Au stade B, intermédiaire entre les précédents, l′infiltrat cellulaire, fait de plasmocytes franchement dystrophiques et d′un petit nombre d′immunoblastes (ou centroblastes) disséminés ou en amas, envahit la sous-muqueuse et parfois la musculeuse.

L′évolution spontanée de l'IPSID peut être continue ou, plus fréquemment, interrompue par des périodes d′amélioration clinique parfois induites par un traitement antibiotique aveugle. Le Traitement à un stade précoce(le stade A) fait appel à une bi antibiothérapie à bas de cycline et de métronidazol pour une durée de six mois la durée de rémission peut aller de 36 mois à 5 ans selon une expérience menée à l'hopital de Saint-Lazare, sur la base d'une étude coopérative tuniso-française [11].

Au stade C et au stade B qui ne répondent pas à l'antibiothérapie, une polychimiothérapie incluant une anthracycline (CHOP) est recommandée. En cas de maladie réfractaire à la polychimiothérapie, une chimiothérapie intensive et une auto-greffe sont recommandées. Notre patient avait un bilan d'extension négatif et a été mis sous chimiothérapie à base de CHOP.

Les patients doivent bénéficier d'une évaluation en cours de traitement, à trois et six mois. Elle doit être clinique, endoscopique (biopsies jéjunales) et biologique (taux sérique des chaînes lourdes alpha). Il n'existe pas de protocole validé pour la surveillance post-thérapeutique, néanmoins elle doit être similaire à celle des autres LMNH (tous les trois mois pendant les deux premières années, tous les six mois pendant trois ans puis une fois par an).un traitement antibiotique et antiparasitaire doit aussi être prescrit, car il peut améliorer le syndrome de malabsorption [11].

## Conclusion

Les IPSID sont une maladie orpheline, plusieurs questions demeurent non résolues et méritent des investigations, notamment des études clinicopathologiques étayant le rôle du C. jejuni et la pathogénie de la progression des IPSID.
